# Association between Periodontitis and Carotid Artery Calcification: A Systematic Review and Meta-Analysis

**DOI:** 10.1155/2021/3278351

**Published:** 2021-09-04

**Authors:** Wenxuan Wang, Zhenghao Yang, Yue Wang, Hongyu Gao, Yan Wang, Qiong Zhang

**Affiliations:** State Key Laboratory of Oral Diseases, National Clinical Research Center for Oral Diseases & Department of Pediatric Dentistry, West China Hospital of Stomatology, Sichuan University, Chengdu, China

## Abstract

Recent studies have supported the relationship between periodontitis and carotid artery calcification (CAC), but still uncertain. This systematic review is aimed at evaluating the association between periodontitis and CAC. The search was conducted in four electronic databases: PubMed, EMBASE, Web of Science, and The Cochrane Library, supplemented by checking references of included articles and related review articles. Eligibility assessment and data extraction were conducted independently. The quality assessment and publication bias analysis were performed. The association between periodontitis and CAC was presented in odd ratio (OR) with 95% confidence interval (CI). Additional outcomes included the percentage of alveolar bone loss in CAC versus non-CAC. Twelve studies were included, and 10 were performed quantity analysis. Periodontitis with secure definition (OR = 2.02, 95%CI = 1.18 − 3.45) and insecure definition (OR = 10.78, 95%CI = 4.41 − 26.34) was associated with CAC. And a higher average percentage of alveolar bone loss (weighted mean difference = 10.84%; 95%CI = 6.40 − 15.48) was also observed in CAC patients compared to non-CAC patients. No significant publication bias was found. The results of this systematic review and meta-analysis revealed a significant relationship between periodontitis and CAC.

## 1. Introduction

Arterial calcification is characterized by the deposition of calcium salts in the arterial wall [1]. These calcifications are considered to be the last step in the development of atherosclerosis and occur in up to 90% of atherosclerosis [2]. Carotid artery calcification (CAC) has been found closely related to cardiovascular disease and cerebrovascular disease [3, 4], which cause approximately 30% of human deaths [5] and are the most common cause of death in industrialized countries [6]. According to research reports, CAC accounts for 30%-70% of coronary artery disease [7] and 75% of stroke [2]. Moreover, the morbidity and mortality of cerebrovascular and cardiovascular diseases are increasing year by year, which imposes a heavy economic burden to families and society [8, 9]. From 2016-2017, the cost of cardiovascular disease in the United States was as high as $36.34 billion, which was 1.5 times that of ten years ago [10]. Therefore, early recognition of CAC is very important. Vascular calcification is a multifactor biomineralization process [11], and its biological mechanism has not been well understood. Risk factors of CAC include age, maleness, high blood pressure, diabetes, and kidney disease [12–14].

Periodontitis is an inflammatory disease caused by a specific bacterial group and is characterized by the destruction of the periodontal ligament or tooth supporting tissue [15]. According to the report of the World Health Organization, about 35-50% of people worldwide suffer from periodontitis [16]. Among them, severe periodontitis is the sixth most common disease in the world, accounting for about 5-15% of the global population and resulting in a global productivity loss of 54 billion US dollars every year [17–19]. Due to the growth of population aging and the significant reduction in tooth loss in the elderly population, the global prevalence of periodontitis is expected to increase in the next few years [19]. Periodontitis plays an important role in the development of various systemic diseases, such as atherosclerosis, stroke, and coronary heart disease [20, 21]. The risk of cardiovascular events in patients with periodontitis is 2-3 times higher than that in the population without periodontitis [22]. The possible pathogenic mechanisms between the two involve direct induction by pathogenic bacteria or indirect induction by inflammatory mediators.

Although recent studies have supported the association between periodontitis and CAC [23–26], no systematic review and meta-analysis confirmed/clarified the association. Studies were selected based on the following PECO outline: population: adults (>16 years old); exposure: individuals with periodontal disease; comparison: individuals without periodontal disease; and outcome(s): any indicator of prevalence and/or levels of CAC. We confirmed the association between periodontitis and CAC, which is the first systematic review and meta-analysis on the relationship between them.

## 2. Material and Method

This systematic review and meta-analysis were conducted following the Preferred Reporting Items for Systematic Reviews and Meta-analysis statement [27] and registered in PROSPERO on 20/03/2021 with ID: CRD42021236977.

### 2.1. Inclusion/Exclusion Criteria

Inclusion criteria were set to search for human prospective and retrospective studies. A study was considered eligible when met the following criteria: (1) randomized controlled trials, controlled trials, cohort studies, case control, and cross-sectional studies; (2) human clinical research; (3) the subject of interest was periodontal disease; (4) studies that assessed the risk estimates of CAC associated with any measure of periodontal status; and (5) only English articles were included.

The following studies were excluded: (1) case report, case series, comments, letters, and abstracts without following publication, (2) animal or in vitro studies, or (3) repetitive studies. Reviews were excluded after checking the references for further manual searches.

### 2.2. Search and Screening

The following four electronic databases: PubMed, EMBASE, Web of Science, and The Cochrane Library were searched up to 02 February 2021 with no year restrictions. The search was limited to English publication. We searched for further publication by checking references of included articles and related review articles.

The search strategies were developed using a combination of medical subject headings (MeSH terms) and free text terms and customized as appropriate for each database. All terms are available in Appendix S1.

Two investigators (WX Wang and ZH Yang) independently assessed abstract, and a third (Y Wang) would check for any difference. If any disagreement, the full text would be screened for inclusion.

### 2.3. Quality Assessment

The quality of cohort studies and case-control studies was assessed by two reviewers independently using the validated Newcastle-Ottawa Quality Assessment Scale, and for cross-sectional studies, the US National Institute of Health (NIH) National Heart, Lung and Blood Institute (NHLBI) quality assessment tool for cross-sectional studies was used. For bias assessment of randomized controlled trials and nonrandomized studies of intervention, the revised Cochrane tool and the ROBINS-I tool would be used. A discussion would be conducted if there was any disagreement with the third reviewer.

### 2.4. Data Extraction

The data of all included studies were extracted by two reviewers independently (WX Wang and ZH Yang). Disagreements were discussed and resolved with a third reviewer (Y Wang). If the effect estimates in publication were not reported, we contacted the authors for more information. A pre-established excel containing the following information was used to extract the relevant data of each study: author, year, study design, population character, diagnostic criteria, exposure definition, and effect (odd ratio (OR)/relative risk (RR) with confidence interval (CI)).

Considering the multiple case definitions of periodontitis found in these literatures, we set two case definitions to assess the methodological quality, adapted from Nibali et al. [28]: (1) *Secure Diagnosis of Periodontitis*. The secure case definition of periodontitis was considered as: at least 2 sites on different teeth with periodontal clinical attachment level (CAL) ≥ 4 mm or 1 site with probing pocket depth (PPD) ≥ 4 mm [29]; generalized chronic periodontitis (at least 30% site with CAL ≥ 4 mm) [30]; at least five sites with CAL ≥ 6 mm [31]. (2) *Insecure Diagnosis of Periodontitis*. The insecure diagnosis of periodontitis was considered as: alveolar bone loss without more clear definition; community periodontal index (CPI) score 3 in at least 1 quadrant; unclear diagnostic criteria for periodontitis or self-reported periodontitis.

### 2.5. Data Synthesis

Statistical analyses were performed using Stata/SE 15.1. The association between periodontitis and CAC was estimated with OR (95% CI). Weighted mean difference (WMD) and 95% confidence interval were calculated for continuous data. For the significant heterogeneity (*P* < 0.1), the random-effects model was applied accordingly to calculate the pooled estimates of effect. The heterogeneity between studies was tested by means of Cochran's test and the *I*^2^ statistic. A sensitive analysis was carried out to assess the stability of the results. Publication bias was analyzed with Harbord test.

## 3. Result

### 3.1. Selection and Characteristic of the Included Studies

**Figure 1:**
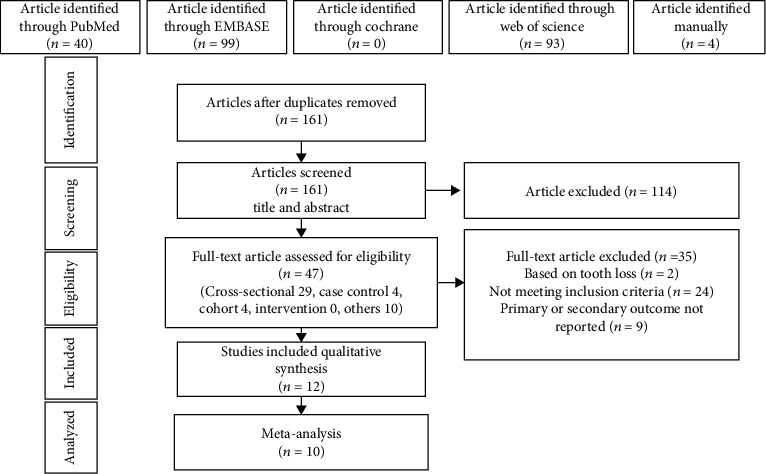
Flowchart of the study selection process.

In the initial research, 40 records were retrieved from PubMed, 99 from EMBASE, 93 from Web of Science, and 4 identified manually. There were 161 records left after removing the duplicates, where 114 of them were excluded while screening titles and abstracts. Then, the full texts of 47 articles were retrieved for further evaluation, of which 35 studies were excluded. Finally, 12 studies were chosen for qualitatively analyses and quantitatively analyses (Figure 1).

Of the 12 articles, 10 publications were included in meta-analyses, all of which are cross-sectional studies, while the other 2 without detailed data suitable for meta-analysis were excluded. The major study characteristics were shown in the evidence tables established according to study design (Appendix S2). In the 12 studies, two were conducted in Japan, three in the USA, three in Turkey, one in Germany, and two in Sweden. As for the study population, all of them enrolled female and male subjects at the same time.

Case definitions for multiple periodontitis were established, and four studies were considered secure and four insecure. Studies have generally reported that the more accepted diagnostic criteria based on digital panoramic radiograph using in the diagnosis of CAC [32–36] or CTs (CBCTs or CTs) [37].

### 3.2. Quality Assessment

The quality of the observational studies we included varied from study to study, while five studies were rated of fair quality, and the other 5 studies were considered as good quality assessed by the US National Institute of Health (NIH) National Heart, Lung and Blood Institute (NHLBI) quality assessment tool for cross-sectional studies (Table 1). The evaluation revealed that the included studies have several potential sources of bias, including case representativeness, sample size justification, or a lack of adjustment for confounding factors. Selection bias and performance bias are the main reasons for the high risk of bias in cross-section studies.

### 3.3. Meta-Analysis

**Figure 2:**
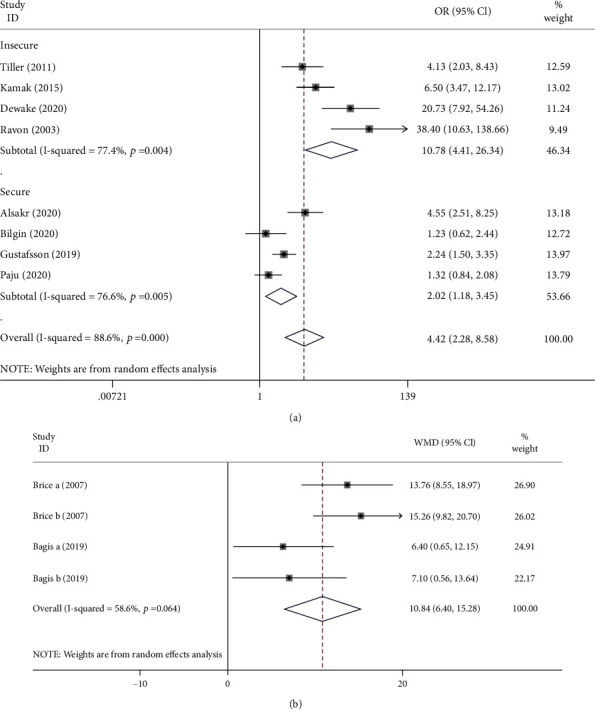
Association between periodontitis and carotid artery calcification. Subgroup analysis forest plots for odd ratio of periodontitis in relationship to carotid artery calcification status in cross-sectional studies. (a) Analysis adjusted for nonconfident definition of periodontitis as described in methods. (b) Analysis for percent alveolar bone loss and carotid artery calcification. The random-effect model was used.

These included studies compared the occurrence of periodontitis in CAC patients with healthy individuals with exposure measure using the periodontal case definition. For the different analyses completed, the *I*^2^ test (CAC 82.7% to 84.5%, PR 77.4% to 76.6%) confirmed statistically significant heterogeneity (Figure 2). Random effect meta-analysis was performed due to the heterogeneity observed in the studies in which odd ratios ranged from 1.23 to 38.40.

Two studies rated periodontitis by the degree of alveolar bone loss with respect to continuous data; therefore, these studies were included in another meta-analysis using inverse variance method (RE) [24, 25].

In 8 cross-sectional studies, the diagnosis of moderate to severe periodontitis was associated with a statistically significant higher occurrence of CAC (OR = 4.42, 95%CI = 2.28 − 8.58; *P* < 0.01). The analysis of two studies using continuous variables predicted the higher levels of alveolar bone loss (WMD = 10.84, 95%CI = 6.40 − 15.28) in patients with CAC compared to non-CAC patients, appearing statistically significant (*P* = 0.008).

Two out of twelve observational studies could not be included in the meta-analysis due to the missing data or different types of outcomes. Bengtsson et al. [26] assessed the presence of carotid artery calcification in periodontitis in an elderly population (60-96 years), indicating a higher prevalence of carotid calcification in individuals with periodontitis (OR = 1.5, 95%CI = 1.0 − 2.3). Ohba et al. [38] evaluated the relationship between periodontal disease and CAC using CPI (Community Periodontal Index) as the periodontal status. However, there was no significant relationship between CPI and CAC with no detailed data.

### 3.4. Subgroup and Sensitivity Analyses

Additionally, sensitivity and subgroup analyses of studies are presented in Table 2. The subgroup analyses were carried out in terms of the definition of periodontitis and the methods used to diagnose CAC. Four studies with secure periodontitis definition confirmed statistical significance (OR = 2.02, 95%CI = 1.18 − 3.45) compared with the meta-analysis of four studies with an insecure periodontitis definition, consistent with the overall result (Figure 2). When comparing the studies diagnosing CAC with CBCTs and CTs vs. panoramic radiographs, the former associations between periodontitis and CAC are considered more significant (OR = 14.14, 95%CI = 3.67 − 54.53, *I*^2^ = 84.5%), but the heterogeneity is higher (Table 2). Three studies reported separately on the prevalence of moderate and severe periodontitis in patients with carotid calcification. Therefore, we performed a subgroup analysis according to the severity of periodontitis, which confirmed that patients with carotid artery calcification present an increased odds ratio (OR = 6.40, 95%CI = 1.03 − 39.78) of diagnosis of severe periodontitis.

Considering the heterogeneity is still relatively high, we made a further sensitivity analysis after the removal of low-quality study (assessed as “fair”) (Table 2). The result of interaction between periodontitis and CAC remains significant with a lower value of heterogeneity (OR = 3.92, 95%CI = 1.98 − 7.73, *I*^2^ = 79.2%, *P* = 0.008).

In addition, the effect of single study on the overall outcomes was assessed by removing a certain study (Table 1). The heterogeneity decreased significantly after the exclusion of Dewake et al. [39] (OR = 3.56, 95%CI = 1.91 − 6.64, *I*^2^ = 86.50%, *P* < 0.01) or Paju et al. [40] (OR = 5.348, 95%CI = 2.28 − 8.57, *I*^2^ = 86.80%, *P* < 0.01).

### 3.5. Publication Bias

**Figure 3:**
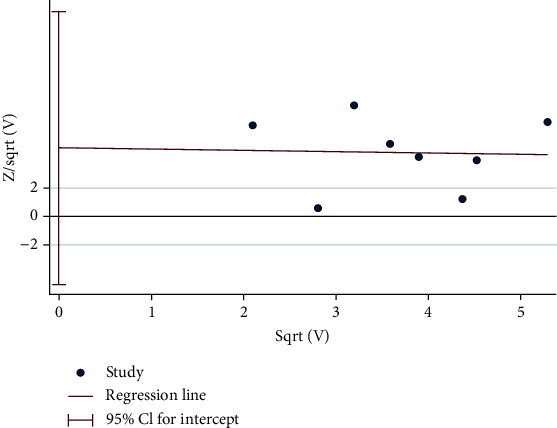
The figure of Harbord test to test publication bias.

Harbord test was used to examine the study publication bias of all the cross-section studies. The Harbord test figure is evaluated visually confirming the studies are symmetric consistent with the Harbord test data, with no statistically significance (*P* = 0.267) (Figure 3). Therefore, there is no suspect of publication bias in this analysis.

## 4. Discussion

The results from the present systematic review support a positive association between CAC and periodontitis. This is the first systematic review and meta-analysis revealing the possible association between the CAC and periodontitis. Based on the quantitative analyses, patients with CAC are more prone to periodontitis than the control group, with an average OR of 4.42 (95%CI = 2.28 − 8.58). In addition, our analysis indicates a positive linear correlation, confirming that patients with CAC are more likely to be diagnosed with severe periodontitis and have alveolar bone loss (10.8%) than those without CAC. This finding is further corroborated in the subgroup analyses with a secure case definition of periodontitis, which still report a significant association with OR of 2.02 (95%CI = 1.18 − 3.45).

Considering the relatively high heterogeneity, we conducted some subgroup and sensitivity analyses to address concerns about the varying quality of studies. The association between CAC and periodontitis remained at a relatively significant level after including only studies with a low risk of bias but with much lower heterogeneity. The same thing occurred when we performed subgroup analysis by including the studies with secured definition of periodontitis, suggesting that bias in observational study designs and inconsistency in diagnostic criteria for periodontitis are the main sources of heterogeneity. More researches with better study designs by standard measurement of periodontal status are required to verify this relationship.

This meta-analysis confirmed an increased occurrence of CAC in patients with periodontitis, which was consistent with the recent clinical and experimental evidence [23–26]. Another experimental animal study employed a variety of approaches and animal models to examine the mutual effect between periodontitis and vascular calcification [41]. In this study, researchers established models for periodontitis, vascular calcification, and periodontitis combined with vascular calcification using Wistar rats, respectively. The ultimate outcomes indicate that not only may periodontitis contribute to vascular calcification, but that vascular calcification could also exacerbate periodontitis.

Besides the experimental result, there are also some researches focusing on the mechanism of correlation between CAC and periodontitis, and taking several conjectures into account is necessary. First, blood lipid is related to the occurrence of periodontitis to a certain extent, leading to hyperlipidemia in which a significant rise of total cholesterol (TC), triglyceride (TG), and low-density lipoprotein (LDL) of patients with periodontitis compared with that of healthy people were found [42–44]. Moreover, this type of association between CAC and periodontitis could be mediated through periodontal tissues induced by *Porphyromonas gingivalis* (*P. gingivalis*) cell wall lipopolysaccharide, which generating degradable apolipoprotein B-100, tumor necrosis factor-a (TNF-a), and interleukin-1*β* (IL-1*β*), causing changes in fat metabolism and eventually leading to the rise of blood lipid once it enters the blood circulation or deep tissue [45].

Second, bacterial factors are also considered in the interaction between CAC and periodontitis. Oral bacteria could enter the bloodstream during periodontal intervention and even during daily oral-hygiene practices, which contributes to transient bacteremia [46]. Using molecular identification methods, Castillo et al. [47] reported a higher possibility (54.8%) of detecting periodontal pathogen in peripheral blood of patients with severity periodontitis after scaling and root planning, compared with 16.6% before the intervention. *P. gingivalis* and *Actinobacillus actinomycetemcomitans* were the pathogens most frequently detected in the peripheral blood before and after periodontal intervention. Some animal studies also demonstrated that the infection of oral pathogens could accelerate aortic atherosclerosis and lipid deposition by intravenous injection of oral bacterial into atherosclerosis-prone animals [48–51]. While Miyamoto et al. [52] and Koizumi et al. [53] reported that preimmunization of *P. gingivalis* could reverse the process of experimental atherosclerosis in the animal model. This interaction was further elucidated that the role in accelerating atherosclerosis may involve oxidative stress, cross-reactive epitopes on bacteria and modified low-density lipoprotein and elevated C-reactive protein in patients with periodontitis [54–56]. Further investigations are desired to clarify the exact mechanism.

Based on this association, due to the preventable and controllable nature of periodontitis, measures of practical operational significance should be taken to maintain periodontal health and reduce the incidence of CAC. Some scholars have proved that hyperlipidemia can accelerate the process of vascular calcification and vice versa, revealing that hyperlipidemia and vascular calcification have mutual promotion and enhancement effects [57]. Furthermore, other studies confirmed that the level of TC, TG, and LDL in patients with periodontitis reduced significantly after periodontal treatment, rising a new idea for postponing the process of vascular calcification and reducing the mortality rate of cardiovascular diseases in clinic [58]. However, the specific pathologic changes and therapeutic principles are still unclear, and more studies are urgently needed.

In view of the high occurrence of CAC in patients with periodontitis and the high sensitivity and specificity of panoramic radiographs detecting CAC [59], it is very economical to diagnose CAC via panoramic radiographs in patients with periodontitis. Moreover, early identification and treatment of CAC can reduce the risk of cerebrovascular and cardiovascular diseases. Therefore, it is an important skill for dentists to diagnose CAC through PR, which requires dentists to be familiar with anatomy and imaging in this area. If the dentist finds CAC via PR during the dental examination, the patient should be referred for cardiovascular evaluation.

This is the first systematic review investigating the possible interaction between periodontitis and carotid artery calcification within the limited scope of observation. However, a number of limitations should be highlighted starting with the limited number of included studies and sample size. Most of these studies are from European and USA, and researches on other regions and ethnicities are few. Moreover, due to the lack of relevant data, we are unable to discuss the influence of other confounding factors, such as gender, smoking, and other systemic diseases, on the association between periodontitis and carotid artery calcification. Meanwhile, the included studies were all cross-sectional studies, and the temporal association between CAC and periodontal disease could not be determined because both were examined at the same time. Without longitudinal studies and intervention studies, there may be some degree of difficulty in establishing a clear causal relationship. Considering the limited value of observational studies, the result of this review requires a more cautious interpretation within the context of the methodology used.

## 5. Conclusion

In conclusion, our data acquired from twelve broad cross-sectional studies and meta-analyses based on it support the hypothesis that periodontal disease is associated with CAC, in addition to the currently known risk factors for CAC, such as hypertension and diabetes. We recommend that future studies use more standard measures to evaluate periodontal status and conduct studies with higher levels of evidence, such as cohort studies and case-control studies. Appropriate sample selection, design, and covariate adjustment should be used to reduce the influence of confounders on the results.

## Figures and Tables

**Table 1:** Quality assessment of cross-sectional studies.

Study	NIH criteria														
	1	2	3	4	5	6	7	8	9	10	11	12	13	14	Quality rating
Tiller, 2011	+	—	NA	—	—	—	NA	—	+	NA	+	—	NA	—	Fair
Kamak, 2015	+	+	+	+	—	—	NA	—	+	+	+	CD	NA	—	Good
Dewake, 2020	+	+	+	—	—	—	NA	—	+	NA	+	CD	NA	—	Fair
Ravon, 2003	+	—	CD	—	—	—	NA	—	+	NA	+	CD	NA	—	Fair
Alsakr, 2020	+	+	+	+	—	—	NA	+	+	NA	+	CD	NA	—	Good
Bilgin, 2020	+	+	+	—	—	—	NA	—	+	NA	+	CD	NA	+	Fair
Gustafsson, 2019	+	+	+	+	—	—	NA	—	+	NA	+	CD	NA	—	Good
Paju, 2020	+	+	+	+	—	—	NA	+	+	NA	+	CD	NA	+	Good
Brice, 2007	+	+	+	+	+	+	NA	+	+	NA	+	CD	NA	+	Good
Bagis, 2019	+	+	+	+	—	—	NA	—	+	NA	+	CD	NA	—	Good

+: yes; -: no; NA: not applicable; CD: cannot determine.

**Table 2:** Subgroup and sensitivity analysis.

	No. of studies	Heterogeneity		Model	Meta-analysis	
		*I*^2^ (%)	*P*		OR	95% CI
Subgroup analysis						
Definition of periodontitis						
Secure	4	76.6	0.005	Random	2.02	1.18-3.45
Insecure	4	77.4	0.004	Random	10.78	4.41-26.34
Diagnosis of CAC						
PR	5	82.7	<0.001	Random	2.46	1.36-4.45
CT/CBCT/DS	3	84.5	0.002	Random	14.14	3.67-54.53
Degree of periodontitis						
Severe	3	95.7	<0.001	Random	6.40	1.03-39.78
Moderate	3	80.6	0.006	Random	2.43	1.04-5.70
Severe-moderate	8	88.6	<0.001	Random	4.42	2.28-8.58

Sensitivity analysis						
Low-quality studies excluded	5	79.2	0.008	Random	3.92	1.98-7.73
Tiller (2011) excluded	7	90.1	<0.001	Random	4.52	2.13-9.55
Kamak (2015) excluded	7	88.9	<0.001	Random	4.19	2.03-8.62
Alsakr (2020) excluded	7	89.9	<0.001	Random	4.47	2.08-9.57
Bilgin (2020) excluded	7	89.9	<0.001	Random	5.34	2.61-10.90
Dewake (2020) excluded	7	86.50	<0.001	Random	3.56	1.91-6.64
Gustafsson (2019) excluded	7	89.8	<0.001	Random	5.05	2.24-11.42
Paju (2020) excluded	7	86.8	<0.001	Random	5.348	2.64-10.81
Ravon (2003) excluded	7	88.6	<0.001	Random	4.42	2.28-8.57

## Data Availability

The data used to support the findings of this study are available from the corresponding author upon request.

## Supplementary Materials

Supplementary 1 Appendix S1: search strategy.

Supplementary 2 Appendix S2: evidence tables.
