# Supplementary material for: Association between Periodontitis and Carotid Artery Calcification: A Systematic Review and Meta-Analysis
Source: Biomed Res Int. 2021 Sep 4;2021:3278351. doi: 10.1155/2021/3278351 (PMC8438587; doi:10.1155/2021/3278351)
Supplement: Supplementary 1 — Appendix S1: search strategy. [file 3278351.f1.docx]

**Appendix S1 Search strategy**

| PubMed | Embase | Web of Science | Central |
| --- | --- | --- | --- |
| #1 (Arteries [MeSH0Terms]  #2 Artery [Title/Abstract]  #3 1 or 2  #4 (Calcification [Title/Abstract]  #5 Calcium [Title/Abstract]  #6 4 or 5  #7 3 and 6  #8 ‘Artery calcification’[Title/Abstract]  #9 ‘Carotid artery calcification’[Title/Abstract]  #10 7 or 8 or 9  #11 Periodontitis [MeSH Terms]  #12 "Periodontal diseases"[MeSH Terms]  #13 "Periodontal diseases"[Title/Abstract]  #14 "Periodontal disease"[Title/Abstract]  #15 "Aggressive Periodontitis"[Title/Abstract]  #16 Periodontitis [Title/Abstract]  #17 "Chronic Periodontitis"[Title/Abstract]  #18 11 or 12 or 13 or 15 or 16 or 17  #19 10 and 18 | #1 Artery/exp  #2 Arter*:ti,ab,kw  #3 1 or 2  #4 Calcification/exp  #5 Calcium/exp  #6 Calcinosis/exp  #7 Calcification: ti,ab,kw  #8 Calcium: ti,ab,kw  #9 Calcinosis: ti,ab,kw  #10 4 or 5 or 6 or 7 or 8 or 9  #11 3 and 10  #12 'Artery calcification':ti,ab,kw  #13 'Carotid artery calcification':ti,ab,kw  #14 11 or 12 or 13  #15 'Periodontitis'/exp  #13 'Periodontal disease'/exp  #14 'Chronic periodontitis'/exp  #15 'Periodontal disease*':ti,ab,kw  #16'Aggressive periodontitis':ti,ab,kw  #17 'Periapical periodontitis':ti,ab,kw  #18 Periodontitis: ti,ab,kw  #19 'Chronic periodontitis':ti,ab,kw  #20 15 or 16 or 17 or 18 or 19  #21 14 and 20 | #1 (Periodontitis)  #2 (Periodontal disease*)  #3 (Aggressive Periodontitis)  #4 (Periapical Periodontitis)  #5 (Chronic periodontitis)  #6 1 or 2 or 3 or 4 or 5  #7 ('Artery')  #8 ('Arteries')  #9 7 or 8  #10 (Calcification)  #11 (Calcium)  #12 (Calcinosis)  #13 10 or 11 or 12  #14 9 and 13  #15 ('Artery calcification')  #16 ('Carotid artery calcification')  #17 14 or 15 or 16  #18 6 and 17 | #1 MeSH descriptor: [Arteries] explode all trees  #2 Arter*: ti,ab,kw  #3 1 or 2  #4 Calcification: ti,ab,kw  #5 Calcium: ti,ab,kw  #6 Calcinosis: ti,ab,kw  #7 4 or 5 or 6  #8 3 and 7  #9 'Artery calcification':ti,ab,kw  #10 'Carotid artery calcification':ti,ab,kw  #11 8 or 9 or 10  #12 MeSH descriptor: [Periodontitis] explode all trees  #13 MeSH descriptor: [Periodontal disease] explode all trees  #14 Periodontal disease*: ti,ab,kw  #15 Aggressive Periodontitis: ti,ab,kw  #16 Periapical Periodontitis: ti,ab,kw  #17 Periodontitis: ti,ab,kw  #18 Chronic periodontitis: ti,ab,kw  #19 12 or 13 or 14 or 15 or 16 or 17 or 18  #20 11 and 19 |
