# Supplementary material for: Association between Periodontitis and Carotid Artery Calcification: A Systematic Review and Meta-Analysis
Source: Biomed Res Int. 2021 Sep 4;2021:3278351. doi: 10.1155/2021/3278351 (PMC8438587; doi:10.1155/2021/3278351)
Supplement: Supplementary 2 — Appendix S2: evidence tables. [file 3278351.f2.docx]

**Appendix S2 Evidence Tables**

| Study | Study design | Country | Population | Diagnostic criteria | Periodontitis definition |
| --- | --- | --- | --- | --- | --- |
| Ohba 2003 | Cross-sectional | Japan | Age: 80  Sex(male/female): 262/397 Smoking: no data  Systemic disease: no data  Sample size(case/control): 33/626 | Panoramic Radiograph | CPI (Community Periodontal Index) |
| Ravon 2003 | Cross-sectional | USA | Age:  DS positive male 69.3±6.7 female 68.3±8.3 DS negative male 59.2±7.6 female 61.3±9.7  Sex(male/female):  DS positive 15/15  DS negative 29/25 Smoking: DS positive nonsmoker 86.2% DS negative nonsmoker 87%  Systemic health: medical record Sample size(case/control): 29/54 | Doppler Sonography | Periodontitis was declared if ≥30% of the teeth had a distance CEJ–BL X4.0 mm. |
| Brice 2007 | Cross-sectional | USA | Age: 52.1 Sex(male/female): 138/63 Smoking: no data Systemic health: Preradiation cancer patient, Sample size: none/unilateral/bilateral: 154/23/24 (CAC) | Panoramic Radiograph | Percent alveolar bone loss |
| Tiller 2011 | Cross-sectional | German | Age CAC 62.34±13.7 NO CAC 46.94±16.14 Sex(male/female) CAC 24/50 NO CAC 325/425 Smoking: no data Systemic health: no data Sample size(case/control): 74/750 | Panoramic Radiograph | Periodontal risk low risk: number of missing teeth ≤ 4, bone reduction index ≤ 0.5,  Moderate risk: number of missing teeth 5 to 8 and/or bone reduction index 0.51-1.0,  High risk: number of missing teeth ≥ 9and/or bone reduction index ≥ 1.1; Bone reduction index: by dividing the maximum bone loss (mm) by the root length |
| Kamak 2015 | Cross-sectional | Turkey | Age  CAC 56.99±8.79 NO CAC 53.35±9.23  Sex(male/female)  CAC 74/82 NO CAC 503/487 Smoking: no data Systemic health: no data Sample size(case/contorl): 156/990 | Panoramic Radiograph | Percent alveolar bone loss: Low periodontal risk: 0-24% |
| Bengtsson 2015 | Cross-sectional | Sweden | Age young-old (60-72) 433 old-old (78-93) 281 Sex (male/female) 186/313 Smoking: no data Systemic health: no data Sample size(case/control): 195/304 | Panoramic Radiograph | Periodontitis was declared if a distance between the alveolar bone level and the CEJ ≥5mm could be identified from the panoramic radiographs presented at >10% of sites, PD of ≥5 mm at one tooth or more and with bleeding on probing at >20% of teeth. |
| Bagis 2019 | Cross-sectional | Turkey | Age CAC ＜50(year old): 43; 50-59: 9;＞59: 8 NO CAC ＜50: 41; 50-59: 13; ＞59: 6 Sex(male/female) CAC 35//25 NO CAC 35/25 Smoking: no data Systemic health: medical record with no systemic diseases Sample size(case/control): 60/60 | CBCT | Percent alveolar bone loss |
| Alsakr 2020 | Cross-sectional | USA | Age: 62±13 Sex(male/female) ICAC(Intracranial carotid artery calcifications) : 53/40 No ICAC: 48/67 Smoking: 22% Systemic health: medical record, especially on CVD  Sample size(case/control): 93/115 | CBCT | Probing depth, gingival recession, clinical attachment loss, bleeding on probing and plaque scores |
| Bilgin 2020 | Cross-sectional | Turkey | Age NO CAC 41.9±15.6 CAC 48±11 Sex(male/female) NO CAC 563/504 CAC 13/21 Smoking: NO CAC 42.9% CAC 50% Systemic health: medical record Sample size(case/control): 34/1067 | Panoramic Radiograph | The patients who had interdental CAL at ≥2 non-adjacent teeth, or buccal or oral CAL ≥3 mm with probing depths >3mm at ≥2 teeth were diagnosed with periodontitis |
| Dewake 2020 | Cross-sectional | Japan | Age  carotid artery calcification 72±9.7 no carotid artery calcification 59.4±10.3 Sex(male/female) carotid artery calcification 68/53 no carotid artery calcification 99/75 Smoking: no data Systemic health: medical record Sample size(case/control): 121/174 | CT | Percent alveolar bone loss |
| Gustafsson 2019 | Cross-sectional | Sweden | Age  CCAA (Calcified Carotid Artery Atheroma) (median): 65  NO CCAA: 63 sex(male/female) CCAA 358/99 NO CCAA 842/183 smoking: no data systemic health: Myocardial Infarction sample size: 457/1025 | Panoramic Radiograph | 1. remaining bone＜80% 2. CPD(clinical periodontitis disease index)≥2; one point was given for each : 1) the presence of dentures, 2) >10 periodontal pockets measuring ≥4 mm, 3) total pathologic pocket depth >37 mm, 4) mean bone loss (based on radiography) of one-third of the bone height or more for all measured teeth, and 5) <16 remaining teeth. |
| Paju 2020 | Cross-sectional | Finland | Age  NO CAC 62.6±9.4 moderate CAC 66.7±7.3 severe CAC 66.5±7.5  Sex(male/female) NO CAC 260/130 moderate CAC 53/28  severe CAC 11/10 Smoking: NO CAC 49.6% moderate CAC 60.5% severe CAC 66.7% systemic health: medical record sample size:  CAC (severe/moderate/no): 21/81/390 | Panoramic Radiograph | Periodontal inflammation burden index (PIBI):[number of 4–5 mm pockets + 2*(≥6 mm pockets)] (Lindy et al. 2008) |
